# Second-generation ICG fusion fluorescence endoscopy in pituitary neuroendocrine tumor surgery

**DOI:** 10.3389/fsurg.2026.1938360

**Published:** 2026-10-21

**Authors:** Huidong Gao, Haoyu Song, Yuyuan Fan, Yongqiang Xu, Zhuoqun Li, Pengfei Liu, Wenbo Gao, Ke Guo, Mengyang Xing

**Affiliations:** 1 Department of Neurosurgery, Binzhou Medical University Hospital, Binzhou, Shandong, China

**Keywords:** delayed imaging, indocyanine green, intraoperative adjunctive technique, pituitary neuroendocrine tumors, second-generation fusion fluorescence endoscopy, transsphenoidal approach

## Abstract

**Objective:**

This study investigated the clinical feasibility of integrating second-generation real-time indocyanine green (ICG) fusion fluorescence endoscopy into routine transsphenoidal surgical workflow for pituitary neuroendocrine tumors (PitNETs). Our focus was on describing the qualitative fluorescence patterns of PitNETs observed within the procedure-embedded 15–18 min delay window following administration of ICG, and evaluating the reproducibility of image interpretation.

**Methods:**

This retrospective single-center descriptive feasibility study included 26 patients with PitNETs who underwent endoscopic transsphenoidal surgery. ICG was intravenously injected before sellar floor drilling, then after the tumor-pituitary interface was fully exposed, real-time fusion mode fluorescence endoscopy was maintained for continuous observation. Two independent observers retrospectively assessed fluorescence intensity and contrast patterns on video clips of intraoperative fusion fluorescence imaging. Interobserver agreement for binary fluorescence intensity grading and three-category contrast pattern classification (tumor-dominant, equivalent, and pituitary-dominant) was evaluated using Cohen's kappa coefficient (weighted kappa for ordinal pattern categories).

**Results:**

Surgery under fluorescence guidance was successfully completed for all 26 patients, with no ICG-related adverse events. No intraoperative mode switching was required, and fluorescence signals remained stable within the delay window. The contrast pattern classifications were: tumor-dominant *n* = 8 (all clinically functioning PRL-PitNETs), equivalent *n* = 15 (multiple subtypes), and pituitary-dominant *n* = 3 (all elderly patients ≥65 years with non-functioning PitNETs). Interobserver agreement was excellent, with Cohen's kappa of 0.91 (95% CI: 0.85–0.97, *P* < 0.001) for binary fluorescence intensity grading and a weighted Cohen's kappa of 0.89 (95% CI: 0.82–0.96, *P* < 0.001) for contrast pattern classification. All postoperative adverse events were mild and resolved conservatively, with no permanent pituitary dysfunction.

**Conclusion:**

Second-generation ICG fusion fluorescence endoscopy demonstrated preliminary technical feasibility and short-term safety for integration into transsphenoidal surgical workflow. The three fluorescence contrast patterns within the 15–18 min window show good interobserver agreement. The clinical utility of these patterns for tumor-pituitary interface assessment remains unproven and requires prospective validation.

## 1 Introduction

Endoscopic transsphenoidal surgery is the main treatment for PitNETs (1). However, under white-light endoscopy, the similar appearance of compressed normal pituitary and tumor tissue makes boundary identification subjective and experience-dependent, contributing to the potential for residual tumor or iatrogenic injury (2, 3). In this respect, ICG fluorescence imaging has been widely applied for intraoperative vascular assessment and tumor boundary identification during neurosurgery (4). Currently, most published studies employed the first-generation fluorescence endoscopes, which required repeated manual toggling between white-light and fluorescence modes, with each switch necessitating refocusing and white-balance calibration. This not only disrupts surgical rhythm and prolongs non-effective operative time, but also fails to provide continuous fluorescence reference during tumor dissection and resection, thereby limiting the widespread application of delayed-window ICG imaging in the routine surgical workflow (5–8). By contrast, the second-generation real-time fusion fluorescence endoscope can superimpose near-infrared fluorescence signals onto white-light anatomical images in real time without requiring manual mode switching, theoretically maintaining the continuity of surgical manipulation and offering better observation for delicate tumor-pituitary boundary dissection (4). To date, most reports on second-generation fusion endoscopy in pituitary surgery have focused on vascular localization. However, systematic data on the qualitative fluorescence patterns of PitNETs and the normal pituitary within the delayed window during routine transsphenoidal surgery remain lacking (9). Furthermore, whether this technique can be fully and seamlessly integrated into standard transsphenoidal surgical workflows without the need for additional waiting time has not yet been clinically validated. Although Lee et al. used fusion fluorescence endoscopy in endonasal surgery, the timing of the observations in their series varied considerably across cases (ranging from 10 to 60 min), without a standardized intraoperative window. Muto et al. proposed the concept of “second-window ICG”, but their study was based on first-generation devices and used a longer observation window, which differs from current routine clinical operative workflows (10, 11).

In comparison, this preliminary descriptive feasibility study examines second-generation real-time fusion fluorescence endoscopy with a core observation window of 15–18 min after administration of ICG, with this delay window being the result of routine bone-manipulation steps in the surgical procedure. The primary objectives were to preliminarily evaluate safety and operability of this technique when it is seamlessly integrated into the standard transsphenoidal surgical workflow. Secondary objectives were to systematically describe the qualitative intraoperative fluorescence patterns of PitNETs and assess interpretative consistency, thereby providing preliminary observational data for future investigations.

## 2 Materials and methods

### 2.1 Patient recruitment

This single-center, retrospective, descriptive study included patients with PitNETs who underwent endoscopic transsphenoidal surgery at the Department of Neurosurgery of our institution between January 2024 and December 2025. The study was approved by the ethics committee of our institution (approval number: LW-126), and all patients provided written informed consent for inclusion in this study preoperatively.

The inclusion criteria included: (1) preoperative imaging and postoperative histopathology confirming a sellar region PitNET; and (2) a surgical approach via the endoscopic endonasal transsphenoidal route. The exclusion criteria included: (1) severe hepatic or renal insufficiency; (2) confirmed allergy to ICG or iodine-based agents after skin testing; (3) pregnant or breastfeeding women; and (4) incomplete clinical, imaging, intraoperative, or follow-up data precluding systematic analysis.

### 2.2 Surgical procedure and ICG administration protocol

All surgeries were performed by the same team of senior neurosurgeons (each with >10 years of experience in endoscopic transsphenoidal surgery) via the standard bi-nostril endoscopic endonasal transsphenoidal approach. ICG (0.3 mg/kg) was injected via an antecubital vein within 30 s prior to commencing sellar floor drilling, and was followed by a 10 mL saline flush. The time required for sellar floor drilling and dural incision served as the ICG distribution period. After full exposure of the tumor-pituitary interface, tumor dissection, resection, and cavity inspection were performed under continuous fusion fluorescence guidance, without requiring manual mode switching or additional waiting time. Resection was carried out along the tumor-normal tissue interface, with the extent adjusted according to the fluorescence signals. The surgical cavity was then re-inspected under fusion mode after the surgeon deemed resection to be complete. Multilayer skull base reconstruction was performed according to the presence of cerebrospinal fluid leakage, followed by routine nasal closure.

### 2.3 Fluorescence imaging system

The fluorescence endoscopic system (NovelBeam Technology, Qingdao, China) employed a single excitation light source peaking at 780 nm, with fluorescence emission spanning 800–840 nm. The endoscope had a 0° viewing angle, a 75° field of view, a diameter of 4 mm, and a length of 18 cm. The system offers white-light, fluorescence, and fusion imaging modes, and in this study all procedures were performed in the fusion mode without intraoperative switching.

Preset parameters, including fluorescence gain, exposure time, and white balance, were fixed for all cases preoperatively and were maintained throughout the procedure to maximize the comparability of fluorescence interpretation across cases. The time from ICG bolus to full interface exposure, which was determined by the surgical procedure steps (sellar floor drilling and dural incision), was recorded for each patient. All fluorescence grading assessments were performed at the time of interface exposure, with the endoscope maintained at a working distance of approximately 2 cm from the tissue surface to ensure a clear field free from significant bleeding or instrument obstruction.

### 2.4 Fluorescence assessment method

Two independent neurosurgeons, blinded to the histopathological results and the patients' clinical information, retrospectively reviewed video clips of intraoperative fusion fluorescence imaging (selecting at least 30 s of stable footage) to perform fluorescence intensity grading and tumor-to-pituitary contrast determination. For this analysis, visible bony structures in the field were used as non-fluorescent background references.

The fluorescence intensity grading criteria were:

Positive (+): Fluorescence signal was bright and obviously higher than the surrounding non-fluorescent background.

Negative (−): No appreciable increase in fluorescence signal, with fluorescence consistent with that of the background.

The tumor-to-pituitary contrast classifications were:

Tumor-dominant pattern: Tumor fluorescence intensity was higher than that of normal pituitary.

Equivalent pattern: Tumor and normal pituitary fluorescence intensities were similar, with no visually appreciable difference.

Pituitary-dominant pattern: The fluorescence intensity of normal pituitary was higher than that of tumor.

The two observers independently assessed each case for both fluorescence intensity grading and fluorescence contrast pattern, with disagreements being resolved by a consensus discussion.

### 2.5 Postoperative assessment criteria

#### 2.5.1 Histopathological subtyping criteria

Tumor subtyping followed the 2022 WHO classification of pituitary neuroendocrine tumors (PitNETs) (12), which distinguishes two related but distinct dimensions: immunohistochemical (IHC) phenotype (adenohypophyseal cell lineage defined by transcription factors and confirmed by hormone protein staining) and clinical functional status (active hormone secretion confirmed by elevated serum levels and corresponding clinical symptoms). A positive IHC result does not always equate to a clinically functioning tumor; silent PitNETs are well-recognized entities, particularly for corticotroph and immature PIT1-lineage subtypes.

The IHC-clinical correspondence for the major PitNET subtypes included in this cohort is summarized as follows:

1. Lactotroph (PRL-lineage): PRL-positive on IHC; most are clinically functioning and associated with hyperprolactinemia, while mild prolactin elevation may also result from stalk effect.
2. Somatotroph (GH-lineage): GH-positive on IHC; most are clinically functioning and associated with acromegaly, but biochemical confirmation is required.
3. Corticotroph (ACTH-lineage): ACTH-positive on IHC; may present as Cushing's disease or as a silent corticotroph tumor.
4. Thyrotroph (TSH-lineage): TSH-positive on IHC; may be associated with central hyperthyroidism, but TSH excess is not exclusive to this subtype.
5. Gonadotroph (follicle-stimulating hormone/luteinizing hormone lineage): Defined by SF1 lineage markers; hormone staining for follicle-stimulating hormone, luteinizing hormone, and *α*-subunit is variable or may be absent. The vast majority are clinically non-functioning.
6. Null-cell PitNET: No detectable lineage-specific transcription factors or hormone expression on IHC; uniformly clinically non-functioning.
7. Plurihormonal PitNET: Immunoreactivity for two or more hormones; clinical function is variable.

#### 2.5.2 Biochemical remission

Biochemical remission criteria for functioning PitNETs were defined based on published consensus and clinical guidelines (13). For PRL-PitNETs, remission was defined as postoperative serum PRL returning to the normal reference range (< 20 μg/L for females and < 15 μg/L for males) without dopamine agonist therapy. For GH-PitNETs, remission was defined as random GH < 1.0 μg/L with age- and sex-normalized IGF-1 levels at 3-month follow-up. For ACTH-PitNETs, remission was defined as early morning serum cortisol < 138 nmol/L (< 5 μg/dL) within 48 h postoperatively, with sustained normalization at 3-month follow-up.

### 2.6 Statistical methods

All statistical analyses were performed using SPSS version 26.0 (IBM Corp., Armonk, New York, USA).

Normally distributed continuous variables are presented as mean ± standard deviation, and categorical variables are presented as frequencies (counts and percentages). The Shapiro–Wilk test was used to verify the normality of data distribution.

Interobserver agreement was evaluated separately for the two retrospective fluorescence assessments:

1. For binary fluorescence intensity grading (positive vs. negative, assessed separately in tumor and normal pituitary regions), Cohen's kappa coefficient was calculated;
2. For ordinal fluorescence contrast pattern classification (tumor-dominant, equivalent, pituitary-dominant), weighted Cohen's kappa coefficient (linear weighting) was applied to account for the graded severity of disagreement between adjacent and non-adjacent categories.

All agreement statistics were calculated from the original independent assessments made by the two observers, prior to consensus discussion. A kappa value ≥ 0.75 was defined as representing good to excellent interobserver agreement. A two-tailed probability of *P* < 0.05 was regarded as statistically significant.

## 3 Results

### 3.1 Patient demographics

A total of 26 patients were enrolled (14 women, age range: 23–70 years, mean 53.3 ± 11.7 years). The postoperative pathological subtypes of the 26 patients were: prolactin (PRL)-PitNETs (*n* = 8), growth hormone (GH)-PitNETs (*n* = 3), adrenocorticotropic hormone (ACTH)-PitNET (*n* = 1), and non-functioning (NF)-PitNETs (*n* = 14). The maximum tumor diameter ranged from 8 to 52 mm (mean 26.6 ± 8.9 mm).

Baseline characteristics by subtype

The PRL-PitNETs had a mean maximum tumor diameter of 26.3 ± 5.1 mm, with Knosp grades of 0–2 in five cases and grade 3 in three cases. The GH-PitNETs had a mean maximum tumor diameter of 20.3 ± 11.0 mm, with Knosp grades of 0–2 in two cases and grade 3 in one case. The ACTH-PitNET had a maximum tumor diameter of 32 mm and Knosp grade of 3. The NF-PitNETs had a mean maximum tumor diameter of 27.8 ± 10.3 mm, with Knosp grades of 0–2 in 11 cases, grade 3 in two cases, and grade 4 in one case. Three of the patients with NF-PitNETs were aged ≥ 65 years. The detailed baseline and fluorescence characteristics for each case are summarized in Table 1.

**Table 1:** Baseline characteristics and fluorescence contrast patterns of 26 PitNET patients.

**Patient**	**Age/ Sex**	**Clinical/biochemical classification**	**Tumor Size (mm)**	**Knosp Grade**	**Preop. Hormone Level**	**Postop. Hormone Level (3 month)**	**Resection**	**Complication**	**Follow-up (months)**	**Tumor fluorescence intensity**	**Pituitary fluorescence intensity**	**Contrast Pattern**	**Time from ICG Injection to Visualization (minutes)**
1	23/F	PRL-PitNET	30	3	PRL: 276.6*μ*g/L	PRL: 12.1μg/L	GTR	None	7	(+)	(−)	Tumor-dominant	16.5
2	63/M	PRL-PitNET	28	2	PRL: 318.4μg/L	PRL: 7.8 μg/L	GTR	None	8	(+)	(−)	Tumor-dominant	17.2
3	55/M	NF-PitNET	21	2	N/A	N/A	GTR	None	8	(+)	(+)	Equivalent	15.7
4	67/F	NF-PitNET	26	2	N/A	N/A	GTR	None	7	(−)	(+)	Pituitary-dominant	16.2
5	60/M	NF-PitNET	29	2	N/A	N/A	GTR	None	6	(+)	(+)	Equivalent	15.8
6	36/F	PRL-PitNET	18	1	PRL: 297.3μg/L	PRL: 16.7 μg/L	GTR	Transient diabetes insipidus	5	(+)	(−)	Tumor-dominant	18.0
7	60/F	NF-PitNET	31	3	N/A	N/A	GTR	None	7	(+)	(+)	Equivalent	17.4
8	54/F	PRL-PitNET	27	2	PRL: 458.2 μg/L	PRL: 10.4 μg/L	GTR	None	6	(+)	(−)	Tumor-dominant	15.2
9	53/M	PRL-PitNET	23	2	PRL: 356.7 μg/L	PRL: 8.1 μg/L	GTR	None	8	(+)	(−)	Tumor-dominant	16.3
10	68/M	PRL-PitNET	31	3	PRL: 459.8 μg/L	PRL: 7.2 μg/L	GTR	Transient diabetes insipidus	6	(+)	(−)	Tumor-dominant	17.5
11	64/M	NF-PitNET	16	2	N/A	N/A	GTR	None	9	(+)	(+)	Equivalent	15.3
12	70/F	NF-PitNET	36	2	N/A	N/A	GTR	None	8	(−)	(+)	Pituitary-dominant	16.7
13	63/F	NF-PitNET	25	2	N/A	N/A	GTR	None	7	(+)	(+)	Equivalent	17.7
14	57/F	PRL-PitNET	32	3	PRL: 516.2μg/L	PRL: 4.9μg/L	GTR	None	9	(+)	(−)	Tumor-dominant	17.3
15	34/F	GH-PitNET	14	1	GH: 31.2μg/L; IGF-1: 756.1 ng/mL	GH: 0.6μg/L; IGF-1: 246.8 ng/mL	GTR	None	10	(+)	(+)	Equivalent	16.8
16	62/M	PRL-PitNET	21	1	PRL: 679.9 μg/L	PRL: 9.7μg/L	GTR	Transient diabetes insipidus	5	(+)	(−)	Tumor-dominant	15.9
17	49/M	NF-PitNET	29	2	N/A	N/A	GTR	None	8	(+)	(+)	Equivalent	16.5
18	40/M	NF-PitNET	33	2	N/A	N/A	GTR	None	7	(+)	(+)	Equivalent	17.1
19	53/F	NF-PitNET	22	2	N/A	N/A	GTR	None	9	(+)	(+)	Equivalent	17.2
20	57/F	GH-PitNET	14	1	GH: 22.3μg/L; IGF-1: 871.1 ng/mL	GH: 0.5μg/L; IGF-1: 194.9 ng/mL	GTR	None	6	(+)	(+)	Equivalent	15.9
21	45/F	ACTH-PitNET	32	3	Cortisol: 38.0μg/dL; ACTH: 190.2pg/mL	Cortisol: 2.8μg/dL; ACTH: 8.1pg/mL	GTR	None	8	(+)	(+)	Equivalent	16.2
22	48/M	NF-PitNET	25	2	N/A	N/A	GTR	None	7	(+)	(+)	Equivalent	16.1
23	50/F	NF-PitNET	8	1	N/A	N/A	GTR	None	8	(+)	(+)	Equivalent	17.3
24	41/F	NF-PitNET	52	4	N/A	N/A	STR	Minor CSF leakage	5	(+)	(+)	Equivalent	16.6
25	65/M	NF-PitNET	36	3	N/A	N/A	GTR	None	9	(−)	(+)	Pituitary-dominant	15.8
26	48/M	GH-PitNET	33	3	GH: 22.6μg/L; IGF-1: 716.7 ng/mL	GH: 0.4μg/L; IGF-1: 240.1 ng/mL	GTR	None	7	(+)	(+)	Equivalent	17.2

Fluorescence intensity (+) indicates bright signal, significantly higher than the non-fluorescent background; (−) indicates no apparent signal. Contrast pattern definitions: Tumor-dominant = tumor fluorescence stronger than pituitary; Equivalent = both intensities are comparable; Pituitary-dominant = pituitary fluorescence stronger than tumor. GTR = gross total resection; STR = subtotal resection; CSF = cerebrospinal fluid; Preop. = preoperative; Postop. = postoperative; N/A = not applicable.

In this cohort, all tumors with PRL, GH, or ACTH immunoreactivity were clinically functioning with confirmed hormonal hypersecretion, whereas all NF-PitNETs showed no biochemical or clinical evidence of hormone excess.

### 3.2 Overall safety and operative performance

All 26 patients underwent successful ICG administration and full-course fluorescence imaging, with no drug-related adverse events being observed. In all cases, no additional mode switching was required during the procedure, with tumor dissection, resection, and cavity inspection all being performed under the fusion field of view, allowing simultaneous acquisition of anatomical information and fluorescence. The surgical workflow remained continuous and uninterrupted, with no additional dedicated waiting time being introduced for the fluorescence imaging. Within the 15–18 min observation window, fluorescence signal intensity remained sufficient for visual interpretation in all cases, with no case requiring termination of observation because of signal loss. The precise interval from ICG administration to full interface exposure for each patient is detailed in Table 1, with a range of 15.2–18.0 min. During the subsequent period from interface exposure to the completion of tumor resection, no significant changes in fluorescence intensity or qualitative reversal of the fluorescence contrast pattern were observed in any case. The contrast relationship between the tumor and the normal pituitary gland remained stable throughout the remainder of the procedure, including during the final cavity inspection after the surgeon deemed resection complete.

### 3.3 Classification of the qualitative fluorescence contrast patterns

Among the 26 patients in this cohort, three major qualitative fluorescence contrast patterns were observed. Detailed fluorescence characteristics are summarized for each case in Table 1.

#### 3.3.1 Tumor-dominant fluorescence pattern

Eight patients exhibited the tumor-dominant fluorescence pattern, characterized by bright fluorescence signal (positive) in the tumor region and no appreciable fluorescence signal (negative) in the normal pituitary region. This results in a visually discernible intensity difference enabling preliminary visualization of the tumor-pituitary boundary under fusion mode (Figure 1).

**Figure 1:**
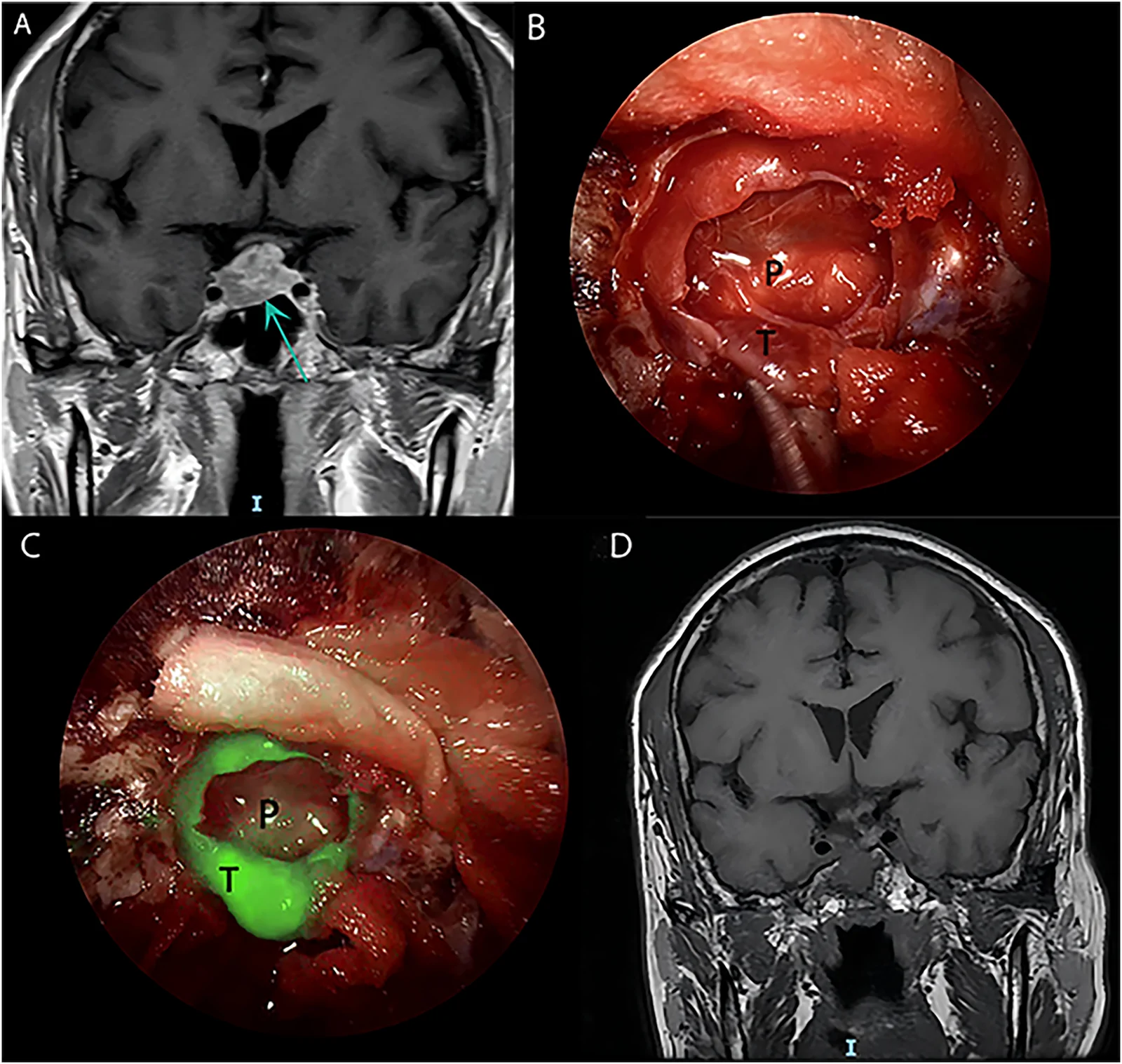
Case 14, a PRL-PitNET. **(A)** Contrast-enhanced T1-weighted imaging shows a relatively clear boundary, with the lesion protruding upward through the septum of the sella turcica and narrowing the right cavernous sinus. It partially surrounds the internal carotid artery and causes a depression at the sellar floor. The arrow indicates the lesion in the sellar region. **(B)** White light image after removing the sellar floor, with the interface between the tumor and the normal pituitary fully exposed. **(C)** ICG fusion fluorescence imaging at 17.3 min after injection, showing the tumor-dominant pattern: the hyperfluorescent area corresponds to tumor tissue **(T)**, while the hypofluorescent area corresponds to the compressed normal pituitary gland **(P)**, the fluorescence intensity in the tumor area is substantially higher than that in the normal pituitary. **(D)** Coronal T1 contrast-enhanced MRI examination performed 3 months after surgery confirming gross total resection.

All eight of the patients with this fluorescence pattern had clinically functioning PRL-PitNETs on postoperative pathology. The mean maximum tumor diameter was 26.3 ± 5.1 mm, and the Knosp grades were 0–2 in five cases and 3 in three cases. The mean observation time from ICG administration to full interface exposure was 16.7 ± 0.9 min.

#### 3.3.2 Equivalent fluorescence pattern

A total of 15 patients exhibited the equivalent fluorescence pattern, characterized by positive fluorescence in both tumor and normal pituitary, with no visually obvious difference in fluorescence intensity.

The patients with this pattern corresponded to the following postoperative pathological subtypes: GH-PitNETs (*n* = 3) (Figure 2), ACTH-PitNET (*n* = 1), and NF-PitNETs in patients aged <65 years (*n* = 11) (Figure 3). The mean observation times were 16.6 ± 0.7 min for GH-PitNETs, 16.2 min for the ACTH-PitNET, and 16.6 ± 0.8 min for NF-PitNETs in patients aged <65 years.

**Figure 2:**
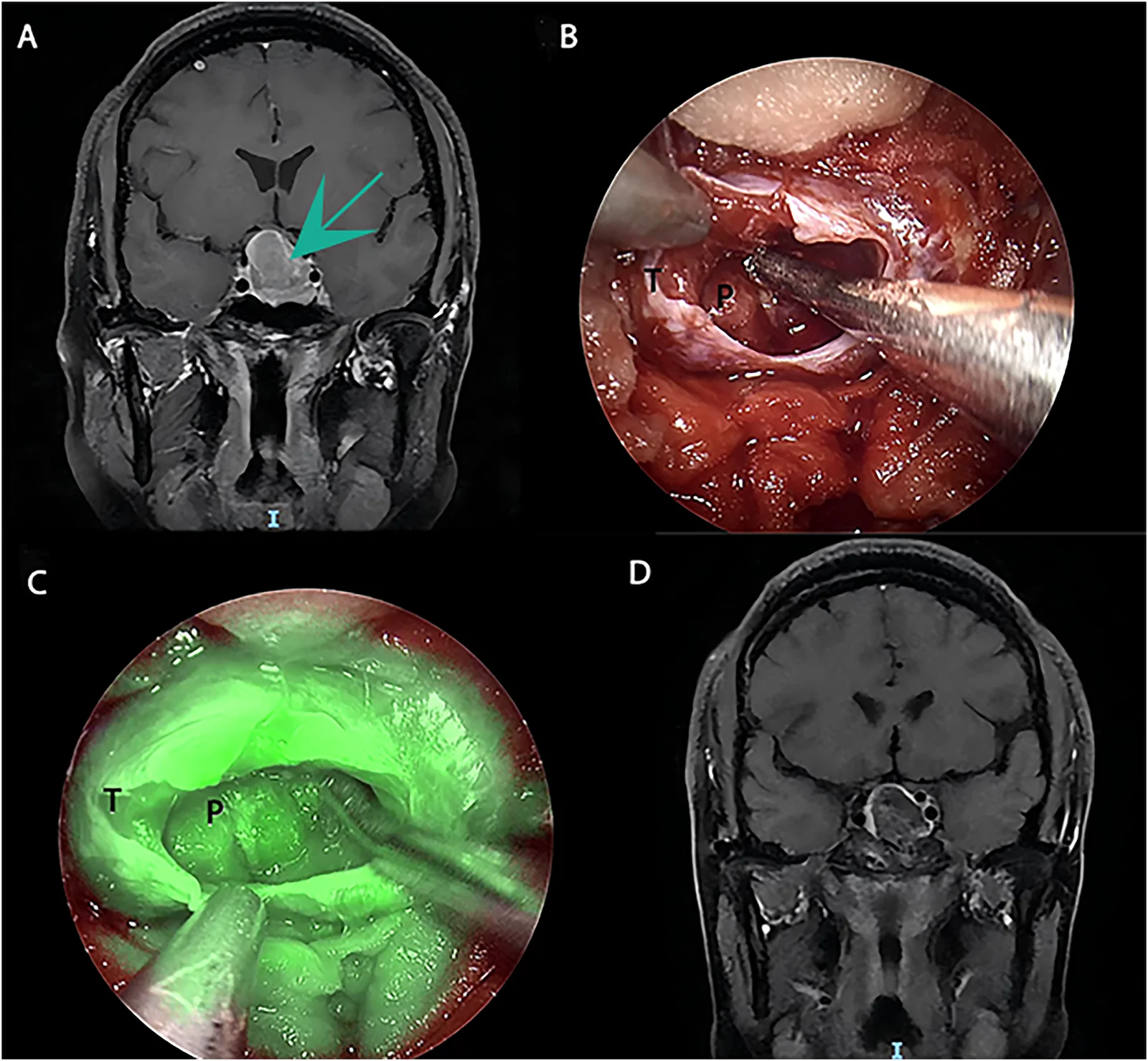
Case 15, a GH-PitNET. **(A)** Contrast-enhanced T1-weighted imaging shows a depression in the pituitary fossa, with the tumor growing towards the right upper direction and the pituitary stalk being displaced upwards. The lesion in the sellar region is marked by an arrow. **(B)** Intraoperative white light imaging after resection of the sphenoid bone shows that the tumor is completely exposed at the junction with the normal pituitary. **(C)** ICG fusion fluorescence imaging at 16.8 min after injection, showing the equivalent pattern: both the tumor tissue **(T)** and normal pituitary gland **(P)** present hyperfluorescence, with comparable fluorescence intensity and no visually discernible contrast difference between the two regions. **(D)** Coronal T1-weighted contrast-enhanced MRI acquired 3 months after surgery confirms gross total resection.

**Figure 3:**
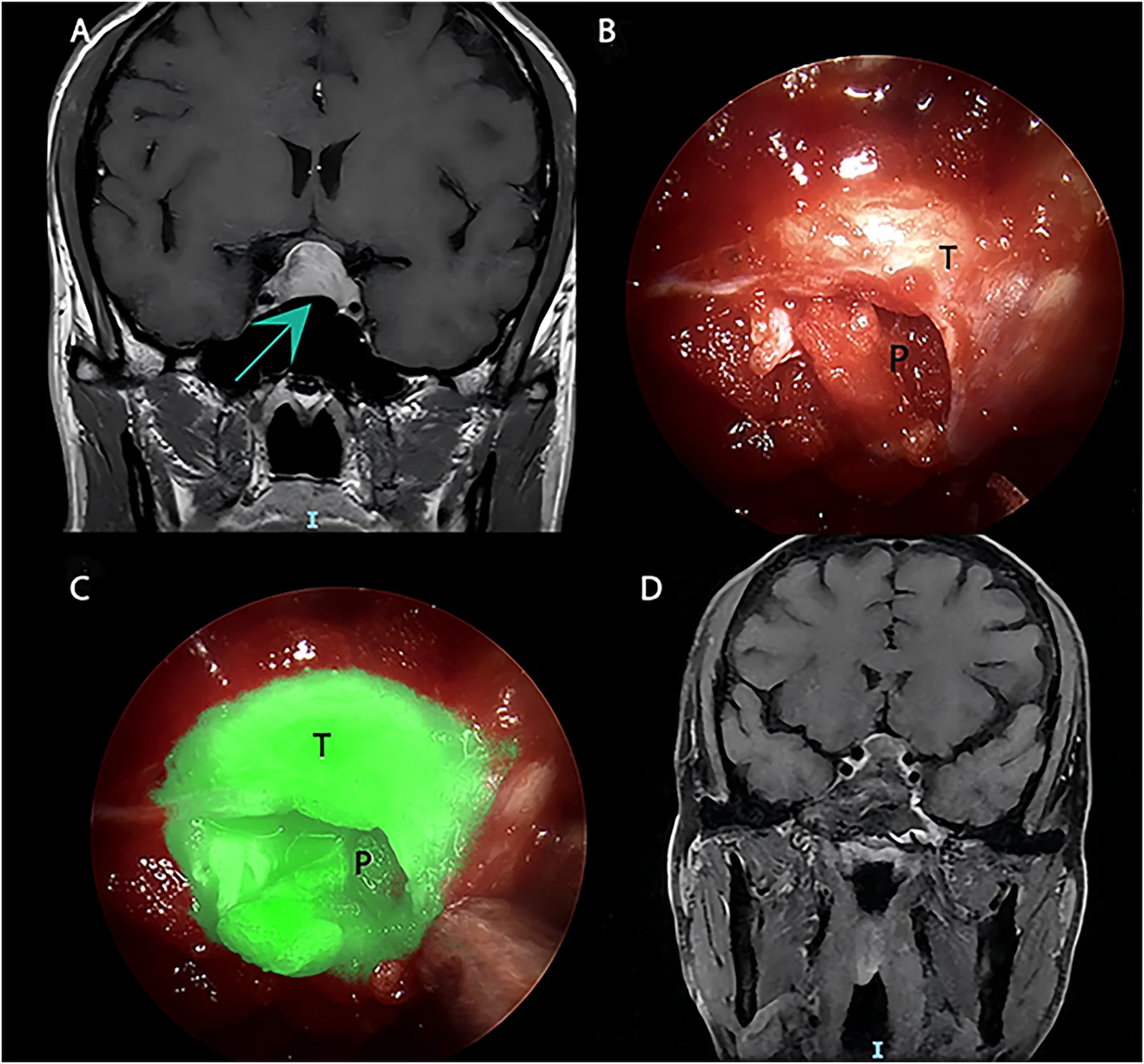
Case 7, a NF-PitNET in a 60-year-old patient. **(A)** Contrast-enhanced T1-weighted imaging shows the lesion compressing the optic chiasm upward, with the pituitary stalk not being clearly displayed. The arrow indicates the lesion area in the sellar region. **(B)** Intraoperative white light imaging after resection of the sellar floor shows the tumor and normal pituitary tissue interface completely exposed. **(C)** ICG fusion fluorescence imaging at 17.4 min after injection, showing the equivalent pattern: both tumor tissue **(T)** and normal pituitary tissue **(P)** present strong fluorescence, with no visually appreciable difference in fluorescence intensity. **(D)** Four months after surgery, coronal T1 contrast-enhanced MRI confirms gross total resection.

#### 3.3.3 Pituitary-dominant fluorescence pattern

Three patients with NF-PitNETs exhibited the pituitary-dominant fluorescence pattern, characterized by bright positive fluorescence in the compressed normal pituitary tissue and no appreciable fluorescence signal in the tumor, with a visually discernible intensity difference enabling preliminary visualization of the normal pituitary boundary under fusion mode (Figure 4). The patients in this subgroup were aged 65, 67, and 70 years. The mean observation time from ICG administration to full interface exposure was 16.2 ± 0.5 min.

**Figure 4:**
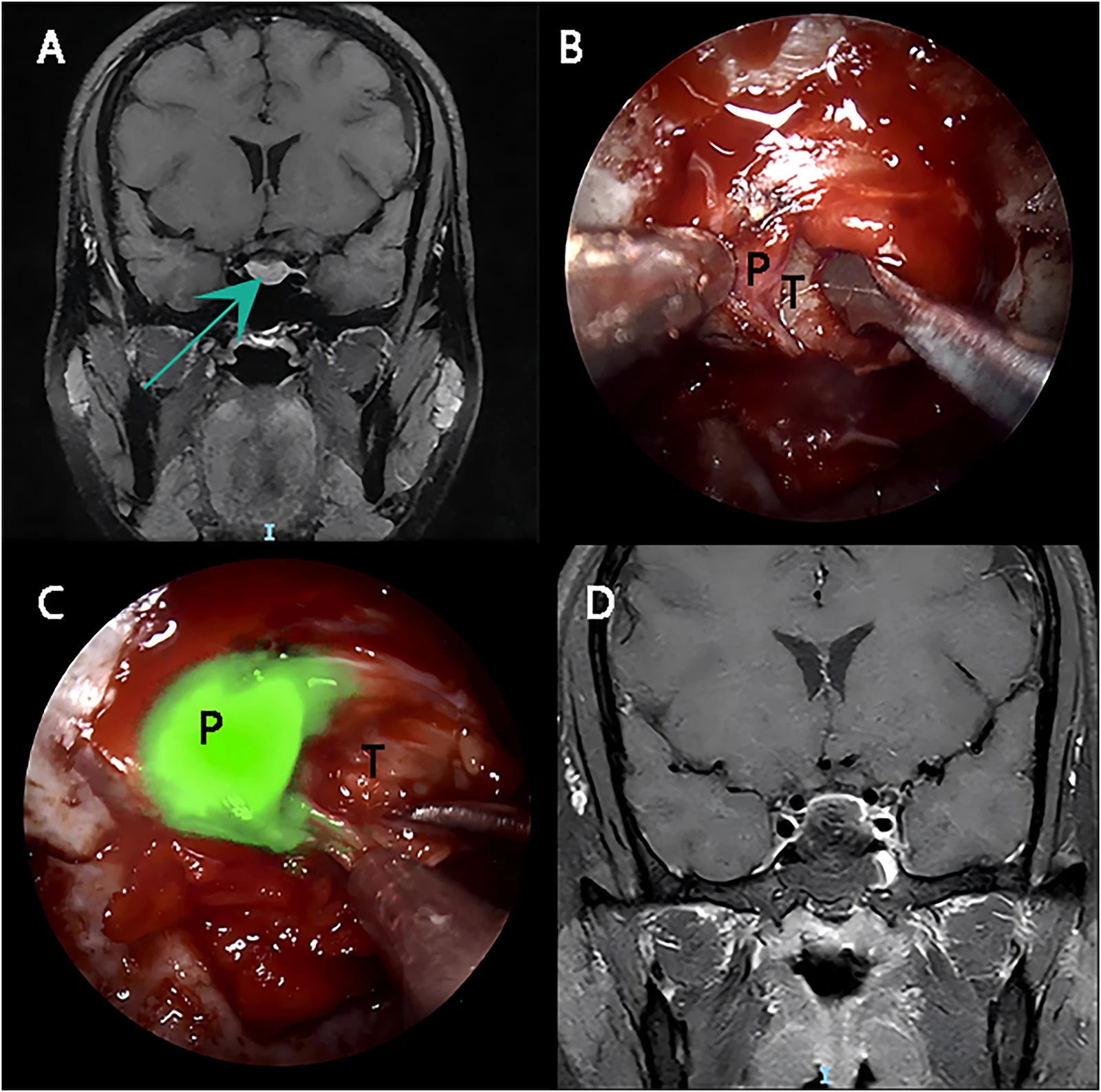
Case 4, a pituitary-dominant NF-PitNET in a 67-year-old patient. **(A)** Preoperative coronal contrast-enhanced T1-weighted MRI shows an enlarged sella turcica, depression of the sella base, a clear tumor boundary, and posterior growth. The arrow indicates the sellar mass lesion. **(B)** White light imaging after removal of the sellar floor shows complete exposure of the interface between the tumor and the normal pituitary. **(C)** ICG fusion fluorescence imaging at 16.2 min after injection, showing the pituitary-dominant pattern: the hyperfluorescent area corresponds to the compressed normal pituitary gland **(P)**, while the hypofluorescent area corresponds to tumor tissue **(T)**, with fluorescence intensity of the normal pituitary substantially higher than that of the tumor. **(D)** Coronal T1-weighted contrast-enhanced MRI acquired 3 months after surgery confirms gross total resection.

### 3.4 Interobserver agreement

For the binary fluorescence intensity grading, the Cohen's kappa coefficient between the two observers was 0.91 (95% CI: 0.85–0.97, *P* < 0.001) for tumor regions and 0.90 (95% CI: 0.83–0.97, *P* < 0.001) for normal pituitary regions. For the ordinal fluorescence contrast pattern classification, the weighted Cohen's kappa coefficient was 0.89 (95% CI: 0.82–0.96, *P* < 0.001). All kappa values exceeded 0.75, indicating excellent interobserver agreement and favorable reproducibility for both of the qualitative fluorescence assessment criteria used in this study.

### 3.5 Surgical outcomes and safety

All patients completed follow-up, with a median duration of 7 months (range: 5–10 months). Among the 26 patients, gross total resection (GTR) was achieved in 25 cases (96.2%), as assessed by the two neuroradiologists on contrast-enhanced sellar magnetic resonance imaging (MRI) performed at 3 months post operation (assessment was made while blinded to all patient details). The single case of subtotal resection was an NF-PitNET with Knosp grade 4 and cavernous sinus invasion, for which postoperative MRI confirmed a minimal parasellar residual tumor.

Among the 8 patients with PRL-PitNETs, the median preoperative prolactin level was 407.5 μg/L (range: 276.6–679.9), and it decreased to 8.9 μg/L (range: 4.9–16.7) at 3-month follow-up. In the 3 patients with GH-PitNETs, the median preoperative growth hormone level was 22.6 μg/L (range: 22.3–31.2), and it decreased to 0.5 μg/L (range: 0.4–0.6) at 3-month follow-up, and the IGF-1 levels of all 3 patients returned to normal. The preoperative morning cortisol of the ACTH-PitNET patient was 38.0 μg/dL, and ACTH was 190.2 pg/mL. At 3-month follow-up, they decreased to 2.8 μg/dL and 8.1 pg/mL, respectively. (Table 1) All patients with functioning PitNETs achieved biochemical remission.

Postoperative adverse events included transient diabetes insipidus in three patients, all of which resolved within 1 week following fluid replacement and desmopressin therapy; and minor cerebrospinal fluid leakage in one patient, which healed after conservative management including bed rest and lumbar drainage. No permanent diabetes insipidus or new-onset pituitary dysfunction was observed during short-term follow-up. No ICG-related adverse events were observed in any of the patients.

## 4 Discussion

### 4.1 Preliminary assessment of workflow integration of the second-generation fusion fluorescence endoscope

ICG is a near-infrared fluorescent dye that is approved by the U.S. Food and Drug Administration (FDA). It is characterized by a favorable safety profile, deep tissue penetration, and low background autofluorescence (14–16). The enhanced permeability and retention effect in tumor microvasculature provides a potential biological basis for ICG-assisted differentiation between tumor and normal tissue (4, 5, 17). The first-generation fluorescence endoscopes previously used in pituitary surgery required manual toggling between white-light and fluorescence modes, with each switch necessitating refocusing and white-balance calibration, which tended to interrupt the surgical rhythm and make it difficult to provide a continuous fluorescence reference during delicate tumor dissection (5–8, 18, 19).

The second-generation real-time fusion fluorescence endoscope employed in this study can superimpose near-infrared fluorescence signals onto white-light anatomical images in real time, thereby enabling simultaneous display of anatomical structure and fluorescence signal on the same screen without the need for manual mode switching by the surgeon. The tumor identification and resection were completed under fusion mode for all 26 patients in this cohort, with a continuous surgical workflow, no intraoperative mode switching, and no device-related adverse events. Therefore, we suggest that this study provides an initial observation of the clinical compatibility of this device with transsphenoidal surgery. Compared with first-generation devices, the fusion mode is better suited for the delicate tumor-pituitary boundary dissection phase in pituitary surgery, providing continuous fluorescence reference without interrupting manipulation, thereby reducing operational disruption and time loss caused by mode switching, and in this manner it aligns more closely with routine clinical surgical workflows.

### 4.2 Fluorescence contrast patterns within the delay window and comparison with the literature

Within the delay window of 15–18 min following ICG administration, three fluorescence contrast patterns were exhibited within our patient cohort: tumor-dominant, equivalent, and pituitary-dominant, with the distribution of these patterns showing both similarities to and differences from previous literature.

The equivalent pattern was the most prevalent in this cohort (15/26), encompassing NF-PitNETs in younger patients (< 65 years), GH-PitNETs, and ACTH-PitNETs. This pattern was characterized by positive fluorescence in tumor and pituitary, with comparable intensities in both. Most previous studies based on first-generation devices reported that the fluorescence intensity of normal pituitary generally exceeded that of tumor within the delay window, although the time-dependent analysis by Muto et al. demonstrated that at 15 min post-administration, the signal ratio between normal pituitary and tumor was only approximately 1.15, and was still in the early phase of rapid contrast ascension (11). The observation timing in this study (15–18 min) falls precisely within this early ascending phase, which likely accounts for the predominance of the equivalent pattern in most cases, rather than a pronounced pituitary-dominant pattern. This finding is consistent with observations from first-generation devices using a similar time window, suggesting that the second-generation fusion endoscope can stably obtain fluorescence imaging of comparable quality to that obtained with conventional devices.

The tumor-dominant pattern was observed in eight cases in this cohort, all of which were PRL-PitNETs. This pattern is characterized by tumor fluorescence substantially stronger than that of normal pituitary. This finding differs from that of previous studies that enrolled PitNETs as a whole without specific subtyping (4). It should be noted that the PRL subtype constituted an extremely low proportion of cases in previous relevant studies; for example, the 25 cases reported by Muto et al. included only 1 PRL-PitNET, meaning that their overall conclusions were primarily driven by GH and NF-PitNETs, and are of uncertain applicability to the PRL subtype (11). However, our study presents a single-center small-sample observation with imbalanced baseline characteristics across groups, and the stability and generalizability of this phenomenon requires validation in larger baseline-matched studies. Whether the consistently hyperfluorescent appearance of PRL-PitNETs observed in this cohort stems from their unique vascular permeability characteristics or distinct ICG clearance kinetics remains unproven.

The pituitary-dominant pattern, characterized by fluorescence of normal pituitary being stronger than that of the tumor, was observed in three cases in this cohort, all of which were patients aged ≥ 65 years with NF-PitNETs (5, 6, 8, 11). This finding is consistent with the late delay-window pituitary hyperfluorescence feature reported in previous studies, but was observed exclusively in the elderly subgroup in this study. The underlying mechanisms may involve age-related alterations in pituitary vascular permeability, remodeling of normal pituitary blood supply due to chronic tumor compression, or age-related differences in ICG pharmacokinetics; the dominant contributing factor cannot be determined at present. Given that this finding is a *post-hoc* exploratory observation in an extremely small number of cases, it should serve only as a hypothesis-generating source for future studies and does not carry definitive clinical guidance implications.

### 4.3 Clinical application and operational recommendations

This cohort observation study suggests that intraoperative interpretation of delayed-window ICG fluorescence is influenced by multiple factors, including the endoscope working distance, bony structures and tissue coverage, intraoperative bleeding, and the positioning of the observation time window. First, previous *ex vivo* experiments demonstrated that ICG fluorescence intensity is optimal at a distance of 2 cm (5). In our cohort, maintaining the endoscope at approximately 2 cm from the tissue surface was critical for obtaining stable signals, with excessive distance leading to signal attenuation, and excessive proximity causing localized halo artifacts that interfered with interpretation. Second, the sellar floor bone thickness and residual arachnoid membrane may affect near-infrared light penetration and signal acquisition (18, 19). Third, local intraoperative bleeding can obscure the true fluorescence signal of target tissues, resulting in false-negative low-fluorescence interpretation, as emphasized in previous reports stating that “ICG observation requires a clear blood-free surgical field” (8, 20). Fourth, the 15–18 min delay window represents the early phase of fluorescence enhancement, and previous pharmacokinetic studies suggest that the signal of normal pituitary is in the ascending phase during this period (11). Although this temporal positioning may influence the absolute signal intensity, the qualitative contrast pattern classification used in this study remained consistent within this window and did not shift with minor time variations.

On the basis of the above limiting factors, ICG fluorescence in PitNET surgery should be positioned as an adjunctive reference to white-light anatomical interpretation, rather than as an independent diagnostic tool. It should be emphasized that although the interobserver agreement of qualitative fluorescence classification was excellent in this study, high agreement only reflects the stability and uniformity of the classification criteria themselves, and does not represent the diagnostic accuracy of fluorescence for differentiating tumor from normal pituitary tissue. Summarizing the findings of this study, we propose the following considerations for clinical application: (1) For patients with preoperatively suspected PRL-PitNETs, delayed-window fusion fluorescence may provide supplementary visual cues for tumor-pituitary delineation, supplementing white-light interpretation. (2) For elderly patients with NF-PitNETs, if a pituitary-dominant fluorescence pattern is observed, it may help identify the contour of compressed normal pituitary and reduce the risk of iatrogenic injury. (3) For the equivalent pattern, fluorescence signals cannot provide effective boundary discrimination, and white-light anatomical landmarks should serve as the primary basis for this. Fluorescence signal interpretation should be integrated with tissue morphology and texture under white light observation, preoperative contrast-enhanced MRI characteristics, and the relative positional relationship between tumor and pituitary during surgery. When fluorescence signals are consistent with white-light morphology, they may reinforce the surgeon's confidence in their interpretation, but when they are discordant, white-light morphology and anatomical landmarks should remain the primary basis for interpretation, and to avoid misinterpretation, fluorescence signals should not be relied upon in isolation. Their clinical value for boundary identification requires further investigation.

### 4.4 Different ICG administration strategies

Currently, three distinct administration strategies exist for ICG application in pituitary surgery. The ultra-early vascular imaging strategy involves an intravenous ICG bolus (conventional dose 0.2–0.3 mg/kg) after sellar dura exposure, with observation of sellar vascular structures within 30–60 s post-injection, and is primarily used for intraoperative localization and protection of critical vascular structures, including the internal carotid artery and cavernous sinus (7, 18). The intraoperative delay-window strategy observes tumor-pituitary interface fluorescence contrast at 15–18 min post-ICG injection, after dural incision, with the aim of assisting in distinguishing tumor from normal pituitary (5, 11). The preoperative second-window ICG strategy involves administering a high-dose of ICG (5 mg/kg) 24 h preoperatively, exploiting non-specific ICG retention in tumor tissue for delayed visualization, and has been reported to achieve favorable tumor visualization in PitNETs (21, 22).

This study adopted an intraoperative delayed-window administration protocol with a dose of 0.3 mg/kg, which is within the same order of magnitude as most intraoperative administration protocols (5–25 mg fixed dose or 0.2–0.3 mg/kg) (4). Compared with the second-window ICG strategy, the advantages of our protocol include: no need for 24-hour preoperative administration, no increase in preoperative waiting time, and a substantially lower drug dose (0.3 mg/kg vs. 5 mg/kg), making it better suited to routine surgical workflows. Combined with the seamless integration capability of the second-generation fusion endoscope, this protocol can provide delayed-window fluorescence reference without altering the standard surgical workflow, thereby offering a lower barrier to clinical translation. Stable fluorescence imaging within the 15–18 min delay window was achieved for all 26 cases included in this study, suggesting favorable operability of this protocol in clinical practice.

### 4.5 Study limitations

First, this is a single-center, retrospective descriptive feasibility study with a limited sample size. The sample sizes of subgroups are too small to define representative subtype-specific fluorescence signatures. Baseline characteristics including tumor size, invasiveness and age are imbalanced across fluorescence pattern groups, and the observed associations between patterns and pathological subtypes cannot be interpreted as causal relationships. The pituitary-dominant pattern in elderly patients is a *post-hoc* exploratory finding without prespecified age stratification, and its generalizability remains unclear. Second, the observation window and assessment method have inherent methodological constraints. The 15–18 min window was determined by surgical procedures rather than a strictly standardized protocol, introducing temporal variability. The study relies on subjective visual grading of fluorescence as positive or negative and on qualitative contrast pattern classification, without quantitative fluorescence analysis (e.g., tumor-to-pituitary fluorescence ratio, signal-to-background ratio, standardized region-of-interest analysis, time-intensity curves); thus, the optimal timing of the delay window cannot be objectively verified. Third, no systematic histological validation of fluorescence-defined boundaries was performed, so the diagnostic accuracy, sensitivity, specificity and predictive value of fluorescence patterns cannot be inferred from the current data. Fourth, the follow-up period was relatively short, with a median duration of 7 months, which may not capture late recurrences or delayed endocrine changes. Additionally, no control group was established, which precludes evaluation of the incremental clinical benefit of fluorescence guidance on surgical outcomes, endocrine function preservation and operative efficiency. The operational advantage of the second-generation fusion system (no mode switching) is only a qualitative clinical observation; the magnitude of workflow improvement remains unproven due to the lack of quantitative endpoints such as time saved, surgeon workload and number of mode switches avoided. Fifth, this technique has inherent limitations in current clinical practice. The predominant equivalent fluorescence pattern provides limited tumor-pituitary contrast, which may restrict its practical utility for intraoperative decision-making. In addition, interpretation of the three fluorescence patterns is experience-dependent, and there is a risk that less experienced or younger surgeons may misinterpret them.

## 5 Conclusions

In this single-center preliminary descriptive feasibility study, second-generation ICG fusion fluorescence endoscopy is technically feasible and shows acceptable short-term safety when integrated into standard transsphenoidal workflow, providing stable delayed-window imaging without additional waiting time. The three fluorescence contrast patterns, characterized as hypothesis-generating findings, had excellent interobserver agreement. However, this study cannot prove that the technique improves tumor-pituitary interface identification, surgical decision-making or endocrine outcomes. Future prospective controlled studies with quantitative fluorescence analysis and histological validation are required to verify its clinical value.

## Data Availability

The original contributions presented in the study are included in the article/Supplementary Material, further inquiries can be directed to the corresponding author/s.
